# Extensive epigenetic reprogramming in human somatic tissues between fetus and adult

**DOI:** 10.1186/1756-8935-4-7

**Published:** 2011-05-05

**Authors:** Ryan KC Yuen, Sarah MA Neumann, Alexandra K Fok, Maria S Peñaherrera, Deborah E McFadden, Wendy P Robinson, Michael S Kobor

**Affiliations:** 1 Department of Medical Genetics, University of British Columbia, Vancouver, BC, Canada; 2 Child & Family Research Institute, Vancouver, BC, Canada; 3 Department of Pathology, University of British Columbia, Vancouver, BC, Canada; 4 Centre for Molecular Medicine and Therapeutics, Vancouver, BC, Canada

## Abstract

**Background:**

Development of human tissue is influenced by a combination of intrinsic biological signals and extrinsic environmental stimuli, both of which are mediated by epigenetic regulation, including DNA methylation. However, little is currently known of the normal acquisition or loss of epigenetic markers during fetal and postnatal development.

**Results:**

The DNA methylation status of over 1000 CpGs located in the regulatory regions of nearly 800 genes was evaluated in five somatic tissues (brain, kidney, lung, muscle and skin) from eight normal second-trimester fetuses. Tissue-specific differentially methylated regions (tDMRs) were identified in 195 such loci. However, comparison with corresponding data from trisomic fetuses (five trisomy 21 and four trisomy 18) revealed relatively few DNA methylation differences associated with trisomy, despite such conditions having a profound effect on development. Of interest, only 17% of the identified fetal tDMRs were found to maintain this same tissue-specific DNA methylation in adult tissues. Furthermore, 10% of the sites analyzed, including sites associated with imprinted genes, had a DNA methylation difference of >40% between fetus and adult. This plasticity of DNA methylation over development was further confirmed by comparison with similar data from embryonic stem cells, with the most altered methylation levels being linked to domains with bivalent histone modifications.

**Conclusions:**

Most fetal tDMRs seem to reflect transient DNA methylation changes during development rather than permanent epigenetic signatures. The extensive tissue-specific and developmental-stage specific nature of DNA methylation will need to be elucidated to identify abnormal patterns of DNA methylation associated with abnormal development or disease.

## Background

The human body contains more than 200 different cell types, each having developed a different function and phenotype despite containing an identical genome. Through the establishment and maintenance of cell type-specific gene expression profiles, epigenetic mechanisms contribute to cellular identity [1]. Perhaps the best understood component of the epigenetic machinery is DNA methylation, which most often occurs on cytosine residues in the context of CpG dinucleotides.

In addition to tissue-specific gene expression, there was a number of intriguing biological phenomena closely linked to DNA methylation, including inactivation of the extra X-chromosome in females [2], allele-specific expression of imprinted genes [3], and biological aging [4,5]. All of these processes are examples of developmental programming of DNA methylation, which are generally considered to be relatively stable. However, recent studies have shown that DNA methylation can be dynamic and capable of temporally changing [6,7]. This plasticity may be modulated in part by a diverse set of environmental influences, all of which have been correlated with changes in DNA methylation. These include nutritional factors such as folate intake [8], social factors such as maternal care [9], and environmental factors such as exposure to pollutants [10,11]. Therefore, it is likely that DNA methylation serves as an important mediator between the environment and genome function. The malleable features of DNA methylation are important for its role in health and disease, as improper regulation of this epigenetic marker during development has been associated with a number of pathological conditions including birth defects and various cancers [12].

One particularly well-understood specialized aspect of epigenetics during development is genomic imprinting, which describes the specific allelic expression, depending on the parent of origin, of a small number of genes. Although this epigenetic program is established early in development and thought to be maintained throughout life [13,14], relatively little is known about its tissue-specific features and temporal dynamics in different developmental stages in humans. In addition to imprinting, a number of findings connecting DNA methylation changes to biological development have emerged over the past few years, largely fuelled by the advent of genome-wide technologies. For example, substantial alterations in DNA methylation occur during stem cell differentiation, supporting a general role for DNA methylation in early development [15-17]. Similarly, profiling of adult human tissues has shown striking differences in DNA methylation, particularly in tissue-specific differentially methylated regions (tDMRs) [18-21]. DNA methylation in adult somatic tissues can undergo striking changes during the adult lifespan, with a tendency for gain of DNA methylation with age for loci (CpG sites) residing within CpG islands (CGIs), and loss of DNA methylation with age for CpG loci residing outside CGIs [22]. It has not yet been determined whether such changes reflect instability in the maintenance of DNA methylation over time, leading to more variable methylation in the older samples, or alternatively, are indicative of intrinsic programmed changes over time, due to changing biological requirements at different developmental and life stages.

It is also not clear to what extent epigenetic programming may be altered by the abnormal development of cells and tissues. Dramatic changes in DNA methylation occur in connection with the altered cellular changes in cancer [23,24]. Reminiscent of cancer, chromosomal trisomy is also associated with altered cell-growth parameters (generally slower growth and increased apoptosis) and a global disruption of the transcriptome [25-27], which could be similarly be associated with altered DNA methylation of a subset of genes. However, comprehensive mapping of DNA methylation has not been performed in subjects with trisomy, especially as it relates to tissue-specific features.

Mechanistically, DNA methylation exerts its effects on gene expression in close partnership with histone proteins [28]. DNA methylation is sensed by proteins that turn on or off gene expression, often through altering post-translational modifications of histones. Numerous histone modifications are associated with different levels of gene expression, most prominently H3K4 trimethylation as an indicator of active transcription, and H3K27 trimethylation as an indicator of inactive genes. Curiously, in stem cells, these markers are sometimes found together in 'bivalent domains', which might poise genes for the rapid expression changes necessary during development [29].

In this study, we investigated the characteristics and functional significance of the differentially methylated CpG loci in normal and abnormal fetal development. Using a well-validated array platform, the DNA methylation status of around 1000 CpG dinucleotides located in the regulatory regions of nearly 800 genes was measured semi-quantitatively in five somatic tissues (brain, kidney, lung, muscle and skin) from second-trimester elective terminations of eight normal, five trisomy 21 (T21) and four trisomy 18 (T18). We found tissue-specific clustering of DNA methylation at this early stage of development in all fetuses, whereas relatively few sites with altered DNA methylation were seen for trisomies. Through a detailed comparison of fetal DNA methylation data with published data on normal somatic tissues from adult autopsies obtained on an identical platform [30], we identified substantial age-related changes in DNA methylation. Lastly, the plasticity of DNA methylation was also evident when we compared fetal DNA methylation profiles to embryonic stem cells [31], with the most variable markers being linked to domains with bivalent histone modifications. Collectively, these data fill an important gap between DNA methylation patterns in stem cells and in adult tissues, and illustrate the complexity that may arise in trying to identify subtler effects of environment or disease.

## Results

### Tissue-specific DNA methylation in fetal tissues

To determine the extent of tissue-specific DNA methylation during fetal development, we used a well-validated array (GoldenGate DNA Methylation Cancer Panel I; Illumina Inc., San Diego, CA, USA) to measure the DNA methylation status in five somatic tissues (brain, kidney, lung, muscle and skin) from second-trimester elective terminations of eight normal, five T21 and four T18 fetuses. For each sample, relative DNA methylation was measured at 1315 CpG loci located in the promoter regions of 752 genes after eliminating probes with a detection *P *value of > 0.05 and those located on the X-chromosome. Only CpG loci located on autosomes were included in the analysis to eliminate gender-specific effects caused by differential methylation of the X-chromosome, which tends to be hypermethylated at gene-regulatory regions in females [32,33].

**Figure 1:**
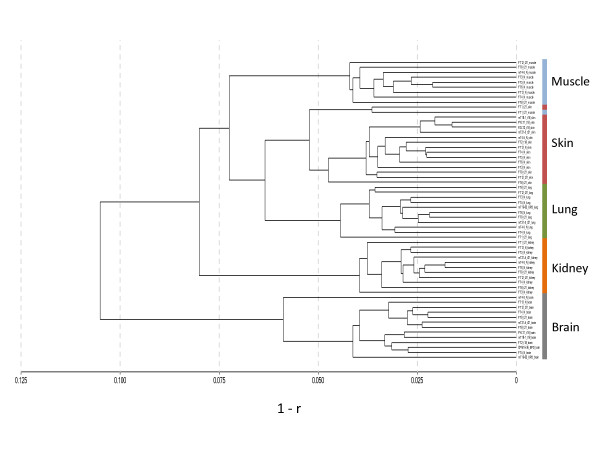
**Unsupervised clustering of fetal tissues demonstrates that each tissue has a distinct DNA methylation profile**. Sample names are shown with labelling of the corresponding tissue types. Tissue samples were clustered by hierarchical clustering of β values based on the formula 1-r (Beadstudio software; Illumina), where r is the correlation coefficient between samples. Specific tissue types clustered together, with strong correlation between samples derived from the same tissue. All tissues had distinct clustering from the other groups, except for one muscle sample from T21 (FT1_t21_muscle), which clustered with the skin sample from the same fetus.

Unsupervised hierarchical clustering (Beadstudio software; Illumina) of the remaining 877 CpG loci was performed based  on the formula 1-r, where r refers to the correlation coefficient between sample methylation values at the included loci. The methylation profiles for samples of the same tissue type were strongly correlated (r > 0.925) and therefore clustered together (Figure 1; see Additional file 1, Figure S1), with the most distinct clustering found for brain. All samples clustered together with strong correlation between samples derived from the same tissue, except for one T21 muscle sample (FT1_t21_muscle), which clustered with the skin sample from the same fetus.

The tight clustering of tissues enabled the identification of CpG loci with tissue-specific DNA methylation profiles. To eliminate potential confounding factors resulting from chromosomal trisomy, this analysis was confined to the five somatic tissues (brain, kidney, lung, muscle and skin) from the eight normal fetuses. Of the 834 sites studied after elimination of invariable sites between normal tissues (see Methods), 195 (23%) were found to be significantly different between tissues as determined by one-way analysis of variance (ANOVA) using a Bonferroni corrected *P *value of 5.99 × 10^-5 ^(see Additional file 2, Table S1).

Of the 195 tDMRs, only 63 (32%) were located within a CpG island (CGI; defined as GC content >50% and observed/expected CpG >0.6 in a length of >200 bp). By contrast, 586 (70%) of the original 834 sites tested were associated with CGIs, suggesting that low-density CpG regions are more likely to dictate tissue-specific DNA methylation patterns (*P *< 0.0001; χ^2 ^test).

**Figure 2:**
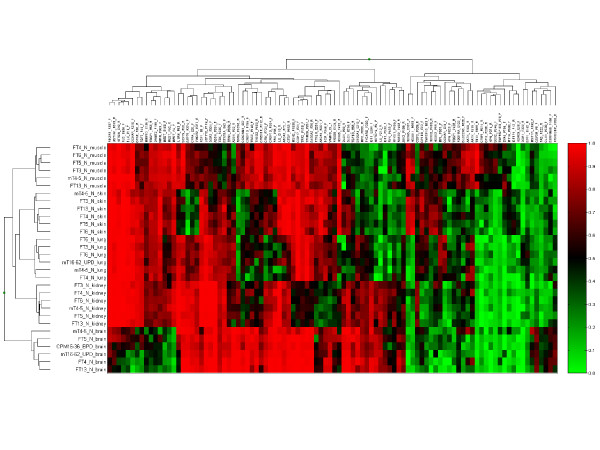
**Heat-map of 98 loci showing hypermethylated or hypomethylated tissue-specific differentially methylated regions (tDMRs) in particular tissues**. Probes and sample names are shown and with hierarchical clustering of β values based on the formula 1-r (Beadstudio software; Illumina), where r is the correlation coefficient between samples. A β value of 0 (bright green) represents an unmethylated locus, and a value of 1 (bright red) represents a methylated locus. Hypermethylated and hypomethylated loci were defined as those having an average β value in that tissue of >0.2 above or below the overall mean for all tissues. Fetal brain had the highest number of tDMRs, with 30 hypermethylated and 23 hypomethylated loci.

To identify the changes that are most likely to be biologically meaningful, we selected for subsequent analysis 98 tDMRs that had an absolute difference in average DNA methylation level for a given CpG site of at least 20% in a particular tissue. Hypermethylated and hypomethylated loci were thus defined as those having an average β value in that tissue of >0.2 above or below the overall mean for all tissues (a β value of 0 represents an unmethylated locus and a value of 1 represents a completely methylated locus). Using this cut-off, fetal brain had the highest number of tDMRs (Figure 2), with 30 hypermethylated and 23 hypomethylated loci, consistent with its more distinct clustering as a separate group (Figure 1). Muscle was the next most distinct tissue, with 24 hypermethylated and 16 hypomethylated tDMRs (see Additional file 2, Table S1).

The ability to identify the tissue source of DNA samples could be useful in determining the developmental origin of pathologically abnormal tissue or other samples of unclear origin. To identify sites that could be used as key indicator markers to identify tissue source, we searched for sites within the fetal tissue data for which the mean of one tissue was maximally different from the mean for other tissues, and in addition, did not show any overlap in the range of DNA methylation values. Using these more stringent criteria, one locus with tissue-specific DNA methylation was identified for each tissue type (five in total: CDH17_E31 for kidney, CRK_P721 for lung, HOXA5_P479 for skin, MUSK_P308 for muscle and MEST_P4 for brain) (see Additional file 2, Table S1). This tissue specificity was confirmed with bisulfite pyrosequencing, with the correlation between values from the microarray and pyrosequencing ranging from r = 0.77 to r = 0.97 (see Additional file 1, Figure S2). These loci are associated with genes (within the promoter region as defined by the Illumina annotation) that are important for the development of their respective tissues [34-36]. For example, MUSK_P208 is associated with the *MUSK *(muscle skeletal receptor tyrosine kinase) gene, which is responsible for synapse formation in mammalian muscle during development [36].

### DNA methylation in somatic tissues from T21 and T18 fetuses had relatively few differences from those of normal fetuses

To identify potential epigenetic differences associated with chromosomal trisomies, the DNA methylation profile in the five somatic tissues (brain, kidney, lung, muscle and skin) from the eight normal fetuses (three male, five female) was compared with the identical tissues from the fetuses with either T18 (n = 4; three male, one female) or T21 (n = 5; two male, three female). Using a cut-off of <5% false discovery rate (FDR) from significance analysis of microarrays (SAM) [37] and a previously suggested Δβ value of >0.17 [38], we identified 17 hypermethylated loci in T18 skin, seven in T21 skin, and one in T21 muscle (Table 1). None of these loci were located on chromosomes 18 or 21. One CpG (DDB2_P407) was hypermethylated in both skin and muscle of T21 fetuses and one CpG (ZNF264_P397) was hypermethylated in T18 and T21 skin (Table 1). No differentially methylated loci were identified in brain, kidney or lung. Furthermore, the tDMRs identified as key indicators of normal fetal tissue types maintained their tissue-specific DNA methylation patterns in the trisomy samples (data not shown). Thus, the significant differences in DNA methylation between chromosomally normal and abnormal fetuses were largely tissue-specific and limited compared with the total number of tissue-specific differences observed.

**Table 1:** Loci showing differential DNA methylation between trisomic and control subjects

					Controls		Trisomies			
Type	Tissue	Feature ID	Chromosome	**FDR**^**a**^**, %**	Mean	SD	Mean	SD	Difference	**GO**^**b **^**term**
T18	Skin	HOXA9_E252_R	7	0	0.04	0.09	0.58	0.21	0.54	Developmental process
		ZNF264_P397_F	19	0	0.52	0.17	0.92	0.01	0.40	Biological regulation
		RYK_P493_F	3	0	0.17	0.08	0.56	0.12	0.39	Developmental process
		CASP10_P186_F	2	0	0.37	0.12	0.72	0.11	0.35	Developmental process
		IL1RN_P93_R	2	0	0.51	0.07	0.78	0.04	0.27	Immune response
		RBL2_P250_R	16	0	0.04	0.05	0.26	0.11	0.22	Biological regulation
		MAP2K6_P297_R	17	0	0.30	0.05	0.49	0.03	0.19	Metabolic process
		JAK3_P156_R	19	0	0.25	0.05	0.44	0.07	0.19	Developmental process
		MST1R_P392_F	3	0	0.15	0.05	0.32	0.05	0.17	Metabolic process
		RARA_P1076_R	17	2.08	0.19	0.08	0.52	0.19	0.33	Metabolic process
		CPA4_E20_F	7	2.08	0.37	0.07	0.61	0.13	0.24	Metabolic process
		CEACAM1_E57_R	19	2.08	0.27	0.05	0.46	0.09	0.19	Developmental process
		ARHGDIB_P148_R	12	3.38	0.74	0.10	0.92	0.02	0.19	Immune response
		SEPT9_P374_F	17	4.24	0.14	0.08	0.37	0.15	0.24	Immune response
		S100A4_E315_F	1	4.24	0.25	0.09	0.43	0.10	0.19	Developmental process
		RAD54B_P227_F	8	4.24	0.16	0.05	0.33	0.12	0.17	DNA repair
		CASP10_E139_F	2	4.24	0.74	0.10	0.92	0.01	0.17	Developmental process

T21	Skin	ZNF264_P397_F	19	0	0.52	0.17	0.85	0.06	0.33	Biological regulation
		WNT10B_P993_F	12	0	0.16	0.04	0.38	0.08	0.22	Developmental process
		DIO3_E230_R	14	0	0.45	0.11	0.65	0.05	0.19	Biological regulation
		TSC2_E140_F	16	0	0.61	0.06	0.79	0.08	0.18	Biological regulation
		IPF1_P750_F	13	3.89	0.33	0.12	0.56	0.12	0.23	Biological regulation
		DDB2_P407_F	11	3.89	0.12	0.09	0.35	0.16	0.23	DNA repair
		HLA-DRA_P77_R	6	3.89	0.70	0.10	0.87	0.05	0.17	Immune response

T21	Muscle	DDB2_P407_F	11	0	0.11	0.05	0.30	0.08	0.19	DNA repair

^a^False discovery rate.

^b^Gene ontology.

### DNA methylation of a significant portion of CpG loci was age-dependent

The establishment of semi-quantitative DNA methylation maps from the fetuses studied allowed us to determine the extent of age-dependent DNA methylation changes. To this end, we compared our data with published DNA methylation measurements obtained from adult human autopsy specimens using the same methylation array [30]. This analysis was limited to the three tissues that overlapped between the two studies (brain, kidney and lung). After eliminating all non-variable CpG loci in the combined fetal plus adult tissue group (β value <0.1 or >0.9 in all samples), 756 loci in brain, 1026 loci in kidney and 849 loci in lung were compared. In general, the average DNA methylation at each autosomal locus in normal fetal tissues correlated more strongly with the average DNA methylation for the corresponding locus in the trisomic fetal tissues (r = 0.99) than the comparable adult tissues (Additional file 1, Figure S3).

**Figure 3:**
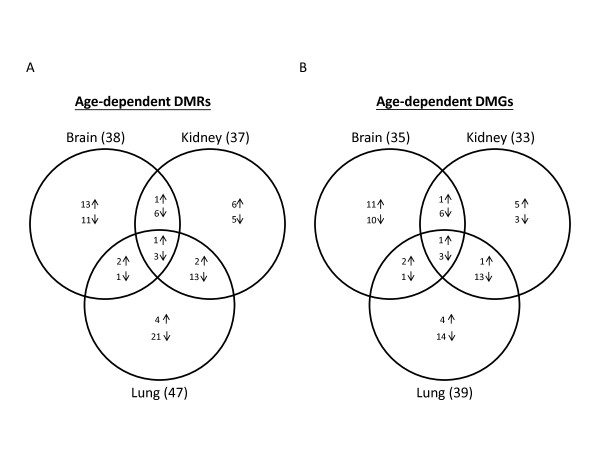
**Venn diagram having the number of age-dependent methylated loci/genes between brain, kidney and lung**. **(A) **Of 89 age-dependent methylated loci (CpG sites), only four loci were common to different tissues. **(B) **Similarly, of the 75 associated genes in the 89 age-dependent methylated loci, only four genes were common to different tissues. Most age-dependent differentially methylated regions (aDMRs) were specific for one tissue, with 24 such loci identified in brain, 11 in kidney and 25 in lung. DMGs = differentially methylated genes. ↑ = Hypermethylated; ↓ = Hypomethylated.

To identify significantly altered sites between fetal and adult tissues, we analyzed raw CpG methylation data using stringent criteria (FDR <5% and Δβ >0.4). This high cut-off for average DNA methylation difference (more than double the suggested β value difference of 0.17) was used to avoid any discrepancies arising from signal differences between arrays due to different hybridization efficiencies or different laboratory facilities performing the experiments [38]. Using this approach, we identified 89 CpG loci, representing 75 distinct genes, for which DNA methylation status was different between fetal and adult tissues. We refer to these as age-dependent (a)DMRs. They represented 10% of the autosomal genes present in the DNA methylation arrays (GoldenGate) used in the two studies (Figure 3A,B; Table 2; see Additional file 2, Table S2). Of these, only four loci (ALOX12_P223, APC_E117, GABRB3_P92 and PEG3_E496) had significant (using our criteria) age-related changes in all three tissues. More commonly, the aDMRs were specific for one tissue, with 24 such loci identified in brain, 11 in kidney and 25 in lung (Figure 3A). Interestingly, these differentially methylated loci included some imprinted regions, such as *GABRB3*, *ZNF264 *and *PEG3 *(see Additional file 1, Figure S4A-C), in which DNA methylation is believed to play a central role in regulating allelic expression in a parent-of-origin manner during normal development [39-41]. There were also many immune-related genes (for example, human leukocyte antigen class II genes) that were hypermethylated in fetal compared with adult tissue, presumably reflecting that the immune system is not yet developed fully in the fetus [42].

**Table 2:** Summary of differentially methylated loci between normal fetal and adult tissues

Tissue	**Number of hyper**^**a **^**loci**	**Number of hypo**^**b **^**loci**	**Total number of aDMRs**^**c**^	Total number of associated genes	**GO**^**d **^**terms**	*P*	**FDR**^**e**^**, %**
Brain	17	21	38	36	-	-	-

Kidney	10	27	37	33	Positive regulation of steroid metabolic process	0.00057	0.87
					Transport	0.0042	4.7
					Regulation of steroid metabolic process	0.0031	4.7

Lung	9	38	47	39	ATP binding	0.0013	1.6
					Positive regulation of steroid metabolic process	0.0013	2
					Positive regulation of lipid metabolic process	0.0015	2.3
					Adenyl ribonucleotide binding	0.0023	2.7
					Transport	0.0026	3
					Adenyl nucleotide binding	0.0028	3.3
					Nucleoside binding	0.003	3.5
					Purine nucleoside binding	0.003	3.5
					Nucleotide-binding	0.0035	4

All hyper^**a,f**^	29	-	29	25	Embryonic morphogenesis	0.0019	2.8

All hypo^**b,f**^	-	60	60	50	NOD^**g**^-like receptor signaling pathway	0.000017	0.018

^a^Hypermethylated.

^b^Hypermethylated.

^c^Age-dependent differentially methylated regions.

^d^Gene ontology.

^e^False discovery rate.

^f^Redundant loci eliminated

^g^Nucleotide-binding oligomerization domain.

**Figure 4:**
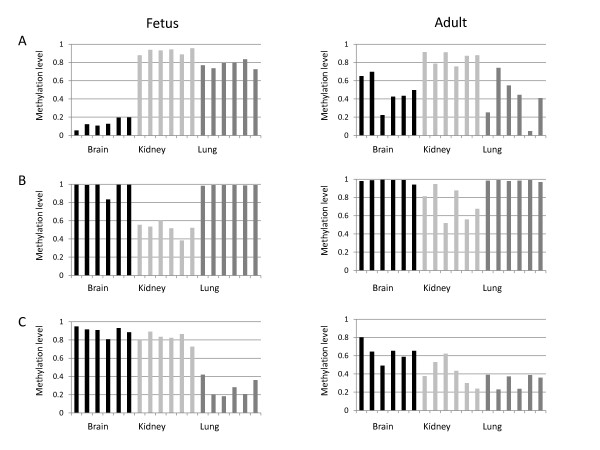
**Lack of conservation in tissue-specific differentially methylated loci between fetus and adult**. Methylation level (β value) of **(A) **MEST_P4 of the *MEST *gene, **(B) **CDH17_E31 of the *CDH17 *gene and **(C) **CRK_P721 of the *CRK *gene in fetal and adult tissues. Each bar represents a different sample. Fetal tissue-specific indicator loci were not indicative of tissue origin in adult tissues.

Together, these data suggest that fetal-to-adult programmed DNA methylation changes occur in a variety of genes within specific tissues. To examine this tissue specificity in more detail, we next focused on comparing tDMRs between fetal and adult tissues. Although a similar number of tDMRs was identified in fetus and adult (93 and 82, respectively), only 25 of these were common to both (see Additional file 2, Table S3). Moreover, of the 25 loci identified as tDMRs in both fetus and adult, only 16 had the same relative tissue-specific DNA methylation pattern in both. Thus, only ~17% (16 of 93) of the fetal tDMRs remained as clear tDMRs in adult tissues. Similarly, 57 tDMRs in adult were not identified as differentially methylated in fetus (see Additional file 1, Figure S5). For example, PTPN6_E171 showed kidney-specific hypomethylation in adult, but was hypomethylated in all the tissues examined (brain, kidney, lung, skin and muscle) in fetus. Furthermore, the fetal tissue-specific indicative loci MEST_P4 (brain), CDH17_E31 (kidney) and CRK_P721 (lung) were not indicative of tissue origin in adult tissues (Figure 4). For example, MEST_P4 was specifically hypomethylated in fetal brain, but all adult tissues had an intermediate level of DNA methylation, consistent with genomic imprinting (Figure 4; see Additional file 1, Figure S4D).

To understand in a developmental context the general function of genes that are differentially methylated between fetus and adult, we carried out a Gene Ontology (GO) analysis using DAVID (Database for Annotation, Visualization and Integrated Discovery; http://david.abcc.ncifcrf.gov/) [43,44]. Thus, we tested whether specific GO terms of the genes associated with one or more aDMRs were enriched compared with the GO distribution of all the 752 autosomal genes associated with CpG sites present on the array that we analyzed. Using this approach, specific GO terms could be assigned to aDMR-associated groups of genes in the three tissues. For brain, there was no GO term enriched for the genes showing age-dependent DNA methylation. For kidney, the enriched function was 'positive regulation of steroid metabolic process' (*P *= 0.00057). Lastly, for lung, the function 'ATP-binding' (*P *= 0.0013) was enriched for the differentially methylated genes. When we did a similar analysis of all aDMRs, irrespective of tissue origin, we found that those genes associated with CpG sites that were hypomethylated in the adult compared with fetus were enriched in the 'nucleotide-binding oligomerization domain (NOD)-like receptor signaling pathway' (*P *= 0.000017), whereas genes associated with hypermethylated sites (increased DNA methylation in the adult) were enriched for 'embryonic morphogenesis' (*P *= 0.0019) (Table 2).

### Characteristics of differentially methylated loci

DNA methylation has been associated with a variety of histone markers and protein-binding targets [15-17]. Understanding how such features are associated with the temporal changes in DNA methylation may provide insight into the regulatory process(es) involved. To test if any chromatin features set up during the embryonic stem (ES) cell stage might affect the fate of tDMRs and aDMRs, we also compared our DNA methylation data with previously published studies of H3K4me3 and H3K27me3 status and Polycomb group (PcG) protein-binding targets in ES cells [45-47].

**Figure 5:**
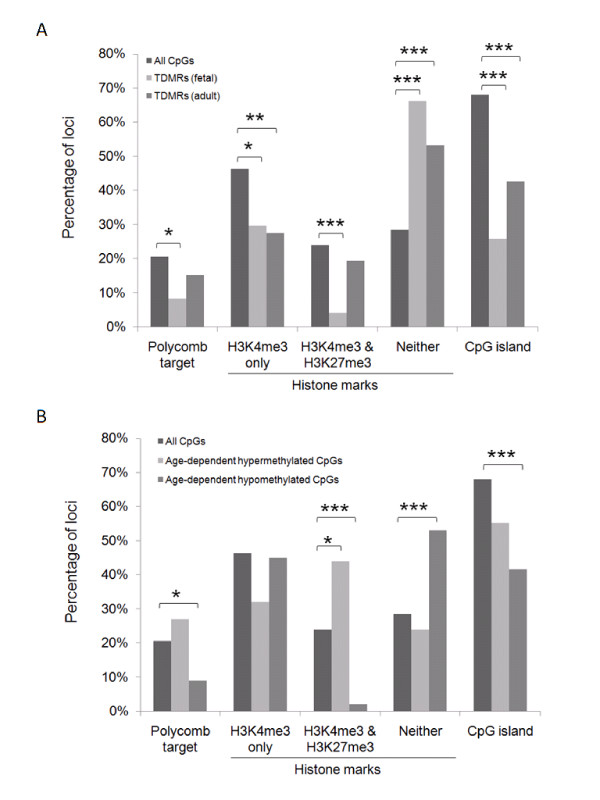
**Characteristics of differentially methylated regions**. Characteristics of **(A) **tissue-specific differentially methylated regions (tDMRs) and **(B) **age-dependent differentially methylated regions (aDMRs). The characteristics of Polycomb complex binding targets and histone markers were based on the previous report on embryonic stem cells whereas the information on CG islands (CGI) was available from Illumina. *P < 0.05, **P < 0.005, ***P < 0.0005. 'Percentage of loci' refers to the percentage of loci in the microarray that contains the specified features.

There were both similarities and differences in the epigenetic marks associated with adult tDMRs compared with those associated with fetal tDMRs. The adult tDMRs were deficient in H3K4me3 regions (*P *= 0.004) and CGI (*P *< 0.0001), but were strikingly enriched in the loci that contained neither H3K4me3 nor H3K27me3 compared with all genes studied (*P *< 0.0001) (Figure 5A), which is consistent with a recent report by Byun *et al*. [30]. Although fetal tDMRs displayed similar characteristics (less prevalent in H3K4me3 regions (*P *= 0.006) and in CGI (*P *< 0.0001), and more prevalent in regions with neither H3K4me3 nor H3K27me3 (*P *< 0.0001)), they were less likely to involve loci containing PcG-binding targets (*P *= 0.006) and regions that were occupied by both H3K4me3 and H3K27me3 ('bivalent' regions) in ES cells (*P *< 0.0001) (Figure 5A).

For the aDMRs, the hypermethylated loci were enriched only in bivalent regions (*P *= 0.03) (Figure 5B), whereas the hypomethylated loci were enriched in regions lacking H3K4me3 or H3K27me3 (*P *< 0.0002) but were reduced in PcG-binding regions (*P *< 0.03), bivalent regions (*P *< 0.0002) and CGI (*P *< 0.0001) (Figure 5B). The reduced number of hypomethylated loci in CGI was also revealed by plotting the DNA methylation distribution of all loci in CGI or non-CGI in fetus and adult independently (see Additional file 1, Figure S6).

### Comparison with ES cells identified dynamic DNA methylation changes

**Figure 6:**
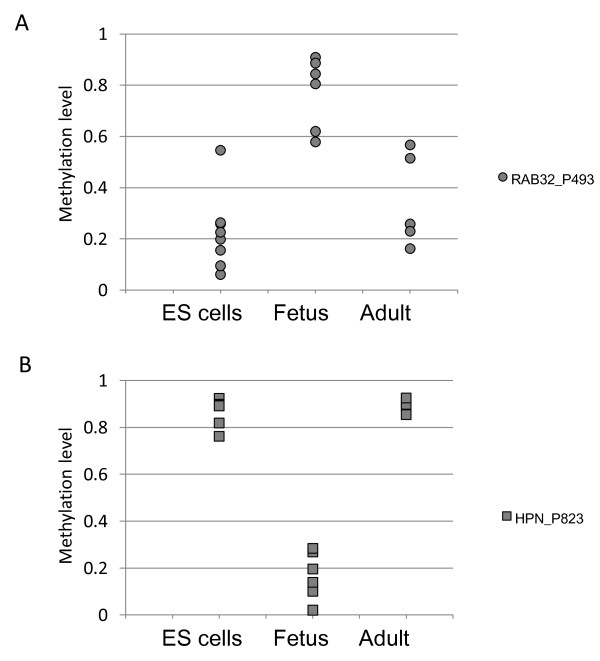
**Dynamic changes of DNA methylation**. **(A) **RAB32_P493 had hypermethylation in fetal brain, but hypomethylation in embryonic stem (ES) cells and adult brain. **(B) **HPN_P823 had hypermethylation in ES cells and adult kidney, but hypomethylation in fetal kidney.

The observed age-dependent DNA methylation changes may represent a distinct temporal program or instead simply reflect a continuum of change from ES cell to fetus to adult. To determine if the fetal DNA methylation profile is largely intermediate between ES cell and adult, we used the same array (GoldenGate Methylation array; Illumina) to compare the identified aDMRs with the DNA methylation pattern of 571 CpG sites in ES cells, obtained from another study [31]. Multiple patterns were seen, with DNA methylation levels at some loci changing dynamically throughout development (Figure 6; see Additional file 1, Figure S7). For example, *RAB32 *was methylated *de novo *in the fetus from ES cells but had loss of DNA methylation in adult tissue (Figure 6A). By contrast, *HPN *had loss of DNA methylation from ES cell to fetus, but was hypermethylated in adult tissue (Figure 6B). This shows that DNA methylation changes dynamically during tissue development.

## Discussion

The establishment and maintenance of tissue-specific gene-expression profiles during development of multicellular organisms is tightly linked to a network of transcription factors and epigenetic modifications. Among the latter, DNA methylation is currently best understood, with a large number of tDMRs having been identified [1,17,18,22,30,48], primarily in adult tissues. In particular, a recent high-throughput DNA methylation study of 11 somatic tissues from six people (aged 35 to 60 years) provided valuable data for adult tissue- and individual-specific DNA methylation patterns [30]. In this study, we present several findings relevant to assessing the contribution of DNA methylation to tissue specificity during the course of normal and abnormal development.

First, we found clustering of fetal tissues according to their DNA methylation patterns, and identified DNA methylation markers that are indicative of tissue origin. Second, although distinct significantly altered DNA methylation markers were present in the skin of fetuses with trisomy 18 and trisomy 21, these differences overall were much less dramatic than tissue- and age-related effects. Third, DNA methylation in adult tissues was remarkably different from that in fetal tissues, with these age-dependent changes most often being tissue-specific. This was also true for imprinted loci, suggesting an unexpected plasticity of these classic epigenetic markers. Lastly, the dynamic nature of DNA methylation markers became even more evident through comparisons with ES cells, with the most plastic regions being linked to bivalent histone-modification domains. Collectively, this work not only complements recent studies identifying DNA methylation changes during aging in blood, but also expands the age range of epigenetic interrogations in somatic tissues, as these have been previously primarily been carried out on adult tissues.

Using an array-based approach, we were able to establish tissue-specific patterns of DNA methylation in fetuses from second-trimester terminations. Unsupervised clustering clearly separated the five tissues examined here, confirming that distinct patterns of DNA methylation occur during early embryonic or fetal development. Consistent with this, 23% of all sites included in the analysis were significantly different between tissues, and were thus classified as tDMRs. Interestingly, tDMRs were more likely to reside in regions of low CpG density as opposed to CGIs, indicating that these regions are particularly receptive for the establishment of tissue-specific DNA methylation markers.

Although fetal tissue-specific DNA methylation was generally maintained in pathological conditions caused by T18 and T21, these chromosomal abnormalities were associated with epigenetic differences. Specifically, we identified 17 hypermethylated loci in the T18, seven in T21 skin, and one in T21 muscle. Interestingly, none of the loci with an altered DNA methylation pattern was located on the affected chromosome (chromosome 18 or 21). This suggests that the extra chromosome may exert a *trans*-acting effect to change the overall epigenetic patterning of the genome, which is consistent with the global disruption in gene expression reported in association with trisomy and in a recent study of genome-wide DNA methylation of leukocytes with T21 [25-27,49]. Many of the differentially methylated genes were related to developmental processes and immune response, perhaps reflecting an important functional difference between normal and trisomic tissues. The lack of obvious DNA methylation differences in brain, kidney and lung between normal and trisomic fetuses may be in part because of our somewhat low sample size (four cases of T18 and five cases of T21) or the relatively small number of CpG loci interrogated here.

In contrast to the relatively subtle changes in DNA methylation associated with the two trisomies, changes in DNA methylation occurring over time in normal development were much more pronounced. In total, 10% of the investigated genes had striking changes in DNA methylation between somatic tissues (brain, lung and kidney) of second-trimester fetus compared with adult. Because we used a high statistical stringency to avoid technical artifacts, even more differences would be expected if less strict criteria were used. Although the cellular composition of each tissue may also change with time, the dramatic differences in DNA methylation between fetus and adult would require major changes in cell composition to explain it. However, it is worth noting that the study was based on the comparison between fetal samples originating from a small time window, with adult samples having a wide age range (age 35 to 60 years), so there is naturally greater variation in the age of adults than in second-trimester fetuses. This may explain the wider variation in DNA methylation seen in adult tissues (Figure 4). A comparison of DNA methylation profiles of adult somatic tissues between two published data sets using the same (GoldenGate) methylation array gave high correlations between the same tissues (r = 0.99 for brain, r = 0.98 for both kidney and lung) [22,30]. In addition, there was at most only one site for each tissue with an average β value difference >0.4 between two data, supporting that the fetal-to-adult difference we found was more profound than can be explained by batch effects. Furthermore, although single nucleotide polymorphisms (SNPs) and sequence repeats overlapping with some probes present on the array may potentially interfere with DNA methylation analysis [30], DNA sequence polymorphisms would be unlikely to cause the consistently large DNA methylation differences seen between groups. In accordance with this, we did not find any enrichment of known SNPs or repeats located in the differentially methylated loci we identified (*P *= 0.92).

Focusing more specifically on the tDMRs that differ between fetal and adult tissues supports the existence of extensive reprogramming of the epigenome occurring during development. Many tDMRs (~80%) identified in fetus were no longer distinctly methylated in the same tissue-specific pattern in adult. This suggests that the tissue-specific DNA methylation, and probably the expression, of these genes is required only at an early stage of development and thus is not maintained in the adult. It is possible that the loss of fetal tDMRs was due either to the reduced function of DNA methyltransferases [50], or to responses to the changing environmental influences, and/or to stochastic changes that occur over time [22]. However, the emergence of some tDMRs in adult that were not present in the fetus suggests that tDMRs also result from major programmed developmental changes occurring postnatally.

One clue to the significance of reprogramming of tDMRs might emerge from the differences in associated biological functions, depending on whether these tDMRs were hypo- or hypermethylated in adult relative to fetus. The age-dependent hypermethylated loci (that is, those that are most probably associated with decreased gene expression in the adult) were enriched for genes involved in embryonic morphogenesis, perhaps reflecting a decreased need for such genes to be expressed in fully differentiated adult tissue. Age-dependent hypomethylated loci were enriched for immune-response pathways, which may reflect the general activation of the immune system after birth.

Mechanistically, chromatin features set in ES cells might be linked to developmental plasticity of tDMRs. Both fetal and adult tDMRs were more prevalent in areas lacking H3K4me3 or H3K27me3, suggesting that tDMRs may be identified by epigenetic markers other than H3K4me3 or H3K27me3. Specifically, tDMRs from fetal tissues were less enriched for bivalent chromatin domains, which are characterized by the coexistence of an activating H3K4me3 marker and repressive H3K27me3 marker. These domains probably function to silence genes encoding developmental regulators, while simultaneously keeping them 'poised' for activation in ES cells [29]. Fetal tDMRs also less often contained PcG protein-binding regions, another hallmark of bivalent domains. PcG proteins are important regulators of cellular development and differentiation [47]. By contrast, there was no significant enrichment of either bivalent chromatin domains or PcG protein-binding regions in adult tDMRs. Together, these findings suggest two conclusions. First, tDMRs present at the fetal stage might regulate processes other than differentiation. Second, the mechanism for tissue-specific regulation of gene expression might differ between developmental stages. However, these conclusions should be taken with caution, given that the actual DNA methylation status of the ES cells being investigated was not taken into account in our study. Further investigation is needed to confirm our conclusions.

These principles are further supported by the observation that CpG loci undergoing DNA methylation changes between fetal and adult tissues often have a distinct DNA methylation pattern in ES cells. For example, whereas it might be expected that *de novo *DNA methylation of genes bound by PcG proteins in ES cells would be irreversible, in order to permanently silence their expression, we in fact found dramatic plasticity at these loci during development. This is well illustrated by *RAB3*2, which had a considerable increase in DNA methylation during the transition from ES cells to fetal brain, but then lost DNA methylation in the adult tissues. Thus, DNA methylation is not only reversible during development, but can be changed in a non-linear, dynamic fashion throughout life. These changes may occur through passive or active processes. These data have important practical implications for DNA methylation studies. Specifically, the developmental plasticity of DNA methylation emphasizes the necessity of using age-matched case-control subjects for epigenetic studies, and considering in what age group the hypothesized differences might be most apparent.

In addition to bivalent chromatin domains being associated with differences between fetal and adult tDMRs, we identified several imprinted loci associated with differential DNA methylation during development. In general, imprinted genes are associated with DMRs that exhibit ~50% DNA methylation, corresponding to their parent-of-origin allelic gene expression pattern. These DMRs are generally classified as either primary (gametic) imprints, which are inherited from the gametes and maintained throughout tissue differentiation, or secondary DMRs, which are generally believed to be acquired before or during tissue differentiation [13]. Although it has been reported that DNA methylation of imprinted genes can change moderately during aging [22,51], and tissue- and developmental-specific imprinting of *Igf2 *has been reported in the mouse [52], this may be more common than previously appreciated.

In this study, we found strong evidence for an erosion of methylation over time for CpGs associated with the promoter regions of several imprinted genes such as *GABRB3 *and *ZNF264*, having an average of ~50% methylation in the fetus, but only ~5% in various adult tissues (for some sites this occurred in all tissues, whereas for other sites, it occurred only in one specific tissue). Interestingly, *GABRB3 *is biallelically expressed in normal brain, including in newborns, but is imprinted in some cases of autistic spectrum disorders [39]. Although we did not measure allelic expression of *GABRB3 *in our fetal samples, the DNA methylation of approximately 50% at the *GABRB3 *locus is indicative of it being imprinted early in fetal development. This then suggests that the early imprinting would have to be erased in brain perinatally to establish biallelic gene expression reported in newborns and adults. Although speculative, it is interesting to consider that autistic disorders might be linked to the maintenance of parent-of-origin allelic expression of *GABRB3*, as a result of failure to erase the fetal imprint.

In addition to loss of imprinting, we also identified gain of DNA methylation at imprinted loci, suggesting that imprinting can be established later in development long after tissue differentiation. For example, *MEST *was unmethylated in fetal brain and highly methylated in fetal lung, but had the expected 50% DNA methylation in both adult brain and lung. Thus, DMRs associated with imprinted genes can not only be tissue-specific but also modulated during the transition from second trimester to postnatal development. This unexpected plasticity raises the possibility that the number of imprinted genes in our genome may greatly exceed those yet identified, as the correct tissue and time point in development may need to be assessed to detect their presence.

## Conclusions

**Figure 7:**
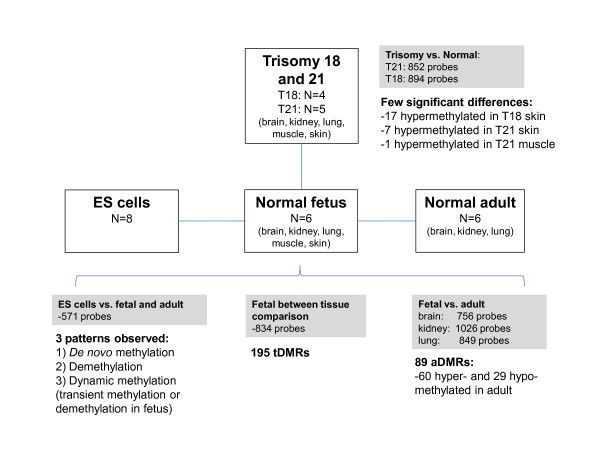
**Summary of data and findings in this study**. Number of probes and tissues is shown for each comparison between sample groups. A brief description of main findings is included.

Although the full biological significance of dynamic changes in tissue-specific DNA methylation over time has yet to be elucidated, the patterns and magnitude of differences indicate many of the changes seen here are programmed rather than stochastic changes (Figure 7). Obtaining well-matched normal human samples over different developmental stages is difficult, thus more detailed investigations in model organisms such as mouse are needed. Nonetheless, the investigation of fetal pathologies such as T18 and T21 cannot be fully replicated in other organisms, and these results suggest that epigenetic changes between disease groups can be identified, as long as there is careful control for all confounding factors such as gestational age and consideration of the effects of tissue composition. These data also suggest that caution should be used in applying DNA methylation analysis to prenatal diagnosis (for example, to diagnose disorders of genomic imprinting) without prior confirmatory studies demonstrating the predictive value of such prenatally determined DNA methylation.

## Methods

The study was approved by the ethics committees of the University of British Columbia and the Children's & Women's Health Centre of British Columbia.

### Sample collection

Fetal tissues (muscle, skin, kidney, lung and brain) were obtained from anonymous, chromosomally normal, second-trimester (15-24 weeks in gestational age, mostly 19-20 weeks) fetuses from elective terminations for medical reasons (that is, termination for premature rupture of membranes or diaphragmatic hernia). Only information on gestational age and reason for pregnancy termination was recorded. All were either dilation or evacuation, with the extractions being of intact fetuses or inductions of labor, which results in delivery of an intact fetus. Samples were collected by the Children's and Women's Pathology laboratory at post-mortem examination as follows: skin (normally abdominal area), kidney (one-quarter of a kidney including the cortex and medulla), brain (cerebrum), lung (small sample from the edges) and muscle (psoas muscle). Genomic DNA was extracted from each tissue sample using standard techniques. Samples from pregnancy terminations for T18 and T21 were obtained in a similar manner for comparison. No discernible growth delay was seen in the trisomic fetuses, and the age distribution was similar for trisomies and controls.

### DNA methylation array

Bisulfite conversion of 750 ng of genomic DNA was performed using a commercial kit (EZ DNA Methylation Kit; Zymo Research, Orange, CA, USA) according to the manufacturer's instructions. After bisulfite treatment, unmethylated cytosines were converted to uracils, whereas methylated cytosines were not changed. Bisulfite-converted DNA samples were subjected to an array-based array (Illumina GoldenGate Methylation Cancer Panel I; Illumina) as described in our previous studies [32,33]. All samples were run on the same chip (GoldenGate; Illumina) to avoid and chip or batch effects. This platform is well-validated, and its use allowed us to compare our data to that in the literature. The array specifically targets promoter regions of the genes (1.5 kb upstream and 500 bp downstream from the transcription start site), and the location of specific sites is well annotated in the probe database (Illumina). Briefly, two allele-specific probes were designed for each CpG site on the array: one for the methylated sequence and one for the unmethylated sequence. After annealing to the target sequence, the probes were extended and ligated to locus-specific oligomers. The ligated products were then amplified by PCR using fluorescently labelled primers, and hybridized to the bead array. The methylation levels for each CpG sites were measured as the intensity of fluorescent signals corresponding to the methylated allele (Cy5) and the unmethylated allele (Cy3). Cy5 and Cy3 fluorescent intensities were corrected independently for background signal and normalized using specialized software (BeadStudio; Illumina). Continuous β values, ranging from 0 (unmethylated) to 1 (methylated) were used to denote the percentage of DNA methylation, from 0% to 100%, for each CpG site. The detection *P *value of each probe was generated by comparison with a series of negative controls embedded in the assay. Probes with a detection *P *value of > 0.05 in any of the samples were eliminated from the study. Furthermore, to concentrate on substantially altered sites and to reduce the statistical complexities associated with large numbers of tests being performed on a small sample set, CpG loci that were considered to be non-variant (β values <0.1 or >0.9 in all samples) were eliminated from the analyses. This was performed throughout the study, but it yielded different numbers depending on the individual comparison, because of the different number of invariant probes in each comparison.

### Statistical analysis

Tissue-specific, differentially methylated regions in fetus and adult tissues were identified by ANOVA for significant CpG loci after Bonferroni correction using statistical software (SPSS; SPSS Inc., Chicago, IL, USA). Loci that were differentially methylated between normal and trisomy fetal tissues, and between normal fetal and adult tissues were identified using SAM, with a cut-off FDR of <5%. Characteristics of DNA methylation in tDMRs and aDMRs were analyzed by the Pearson χ^2 ^test. The Pearson linear correlation was used to analyze the similarities between tissue samples of average DNA methylation at each autosomal locus. The DAVID program was used for GO analysis [43,44]. Using the total number of genes presented in the array as a background for comparison, enriched GO terms were identified using a cut-off FDR of <5%.

### Bisulfite pyrosequencing

Loci identified with tissue-specific DNA methylation in fetal tissues were confirmed using bisulfite pyrosequencing, performed on a pyrosequencer (Pyromark™ Q96 MD; Biotage, Uppsala, Sweden) and the quantitative levels of methylation for each CpG dinucleotide were evaluated with the manufacturer's software (Pyro Q-CpG software; Biotage). DNA methylation-unbiased pyrosequencing primers covering the same CpG sites interrogated by the Illumina probes and their assay conditions are listed (see Additional file 2, Table S4).

## Competing interests

The authors declare that they have no competing interests.

## Authors' contributions

RKCY, WPR and MSK conceived and designed the study. RKCY, SMAN, AKF and MSP performed the experiments. RKCY, MSK and WPR analyzed the data. DEM contributed the fetal tissue samples. RKCY, WPR and MSK drafted the paper. All authors read and approved the final manuscript.

## Supplementary Material

Additional file 1 Figure S1 to S7 Click here for file

Additional file 2 Table S1 to S4 Click here for file
