# Supplementary material for: Extensive epigenetic reprogramming in human somatic tissues between fetus and adult
Source: Epigenetics Chromatin. 2011 May 5;4:7. doi: 10.1186/1756-8935-4-7 (PMC3112062; doi:10.1186/1756-8935-4-7)

Supplementary Figure Legends.

Supplementary Figure 1. Heat-map of the methylation array data. Hierarchical clustering of CpGs (columns) and samples (rows) is based on 1-r of the β values (Illumina Beadstudio software). A beta value of zero (indicated in bright green) represents an unmethylated locus and one (indicated in bright red) represents a methylated locus.

Supplementary Figure 2. Correlations of DNA methylation measurements between Illumina methylation array and bisulfite pyrosequencing. Methylation level (β value) measured by Illumina methylation array is plotted against methylation level measured by bisulfite pyrosequencing for (A) CDH17_E31, (B) CRK_P721, (C) HOXA5_P479, (D) MEST_P150 and (E) MUSK_P308. Linear trendline and R^2 are shown for each comparison. Values for all 5 loci are significantly correlated ($p<0.005$).

Supplementary Figure 3. Correlations of average methylation β values between different tissues. The correlation coefficients between paired tissues are indicated and can range from 0 (yellow) to 1 (blue). Boxes highlighted in red indicate the comparisons between the same tissue type by comparing control to trisomic tissues, or fetal to adult tissues. Trisomic and chromosomally normal fetal show high correlation relative to the same tissue at different developmental time points.

Supplementary Figure 4. Graphs representing different patterns of age-dependent differentially methylation at sites associated with imprinted genes. Average methylation level (β value) is given for (A) GABRB3_P92, (B) ZNF264_P397 in ES cell, fetal and adult tissues, (C) PEG3_E496 and (D) MEST_P4 in fetal and adult tissues. In some cases changes occur in different tissues concordantly over time (e.g. GABRB3_P92_F and PEG_E496_F) while for others the changes are tissue specific (e.g. MEST_P4_F).

Supplementary Figure 5. Lack of conservation of tissue-specific differentially methylated loci in fetus and adult. Methylation level (β value) of (A) FGF1_P357 (B) PTPN6_E171 (C) MST1R_E42 in fetal and adult tissues is given. Each bar represents a different sample. Hypomethylation of FGF1_P357 in brain and MST1R_E42 in lung is specific to adult tissue, whereas hypomethylation of PTPN6_E171 observed in adult kidney represents the fetal status.

Supplementary Figure 6. DNA methylation distribution of all CpG loci in fetal and adult tissues. The number of CpG sites (y-axis) for a given methylation range (X-axis) is

given for brain, kidney and lung considering (A) non-CpG island in fetus, (B) non-CpG island in adult, (C) CpG island in fetus and (D) CpG island in adult.

Supplementary Figure 7. Patterns of DNA methylation changes from ES cell to adult tissues. Examples are given of loci in different tissues that show either *de novo* methylation in adult tissue as compared to fetus and ES cell, demethylation in adult tissue as compared to fetus and ES cell or dynamic (changing) methylation pattern from ES cell to adult tissues. Each data point is an average of the methylation values observed for that site in either ES cell, fetal, or adult samples.

Supplementary Figure 1

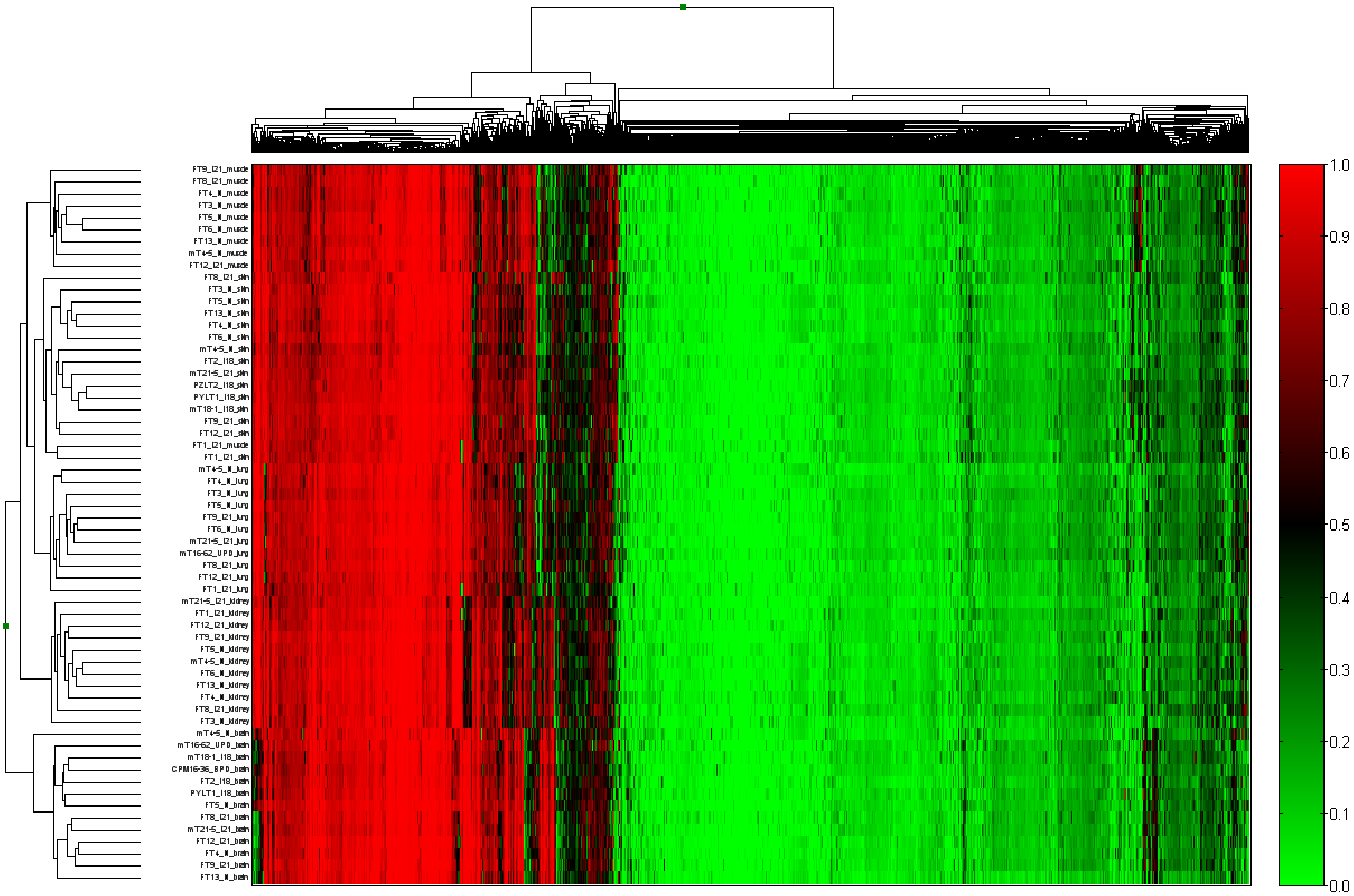

Supplementary Figure 2

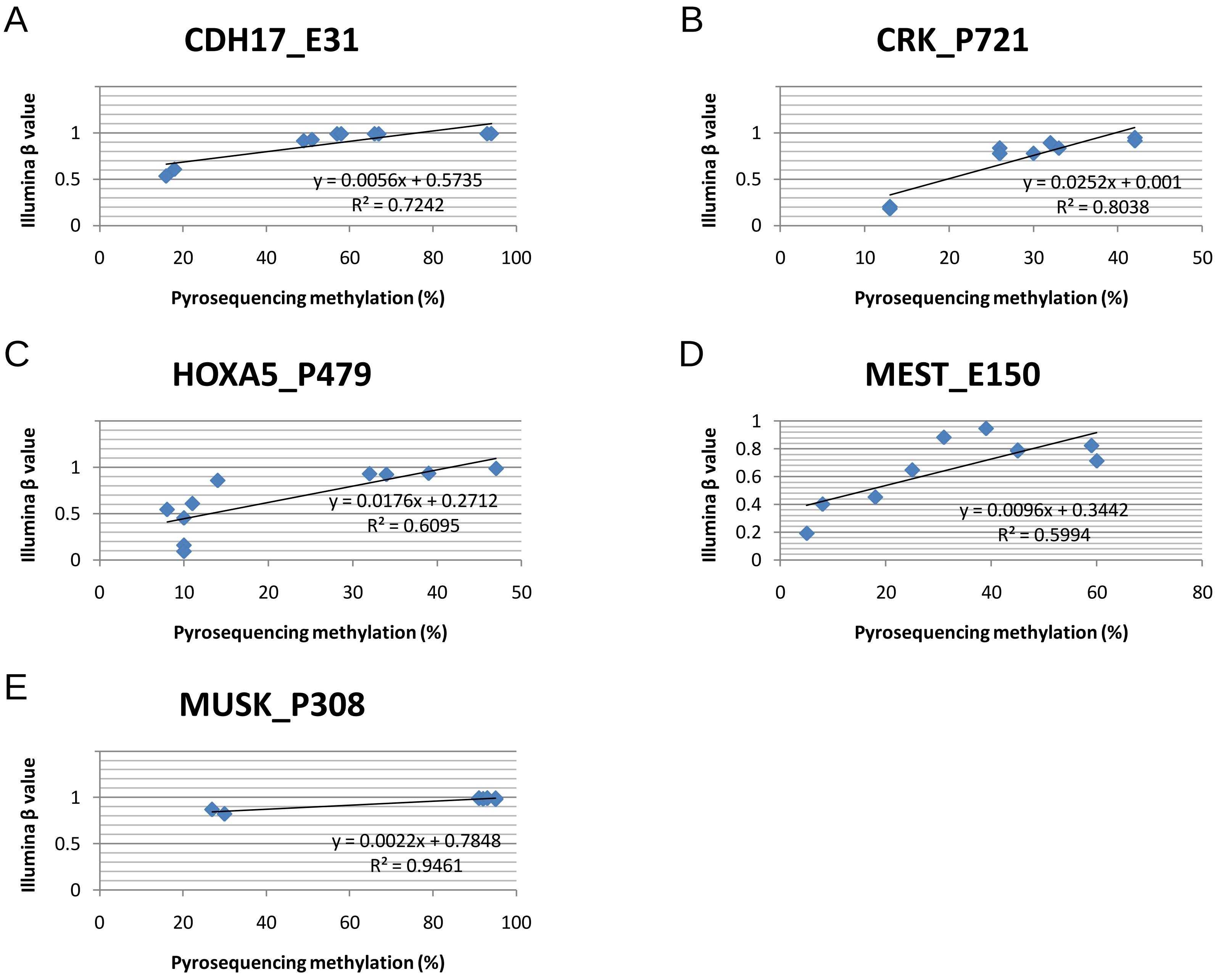

Supplementary
Figure 3

	Fetal Brain	Fetal Kidney	Fetal Lung	Trisomy Brain	Trisomy Kidney	Trisomy Lung	Adult Brain	Adult Kidney	Adult Lung
Fetal Brain	------------	0.96	0.95	0.99	0.96	0.95	0.92	0.89	0.86
Fetal Kidney		------------	0.97	0.94	0.99	0.96	0.90	0.92	0.88
Fetal Lung			------------	0.93	0.97	0.99	0.91	0.92	0.91
Trisomy Brain				------------	0.94	0.94	0.89	0.86	0.83
Trisomy Kidney					------------	0.97	0.90	0.92	0.88
Trisomy Lung						------------	0.91	0.92	0.90
Adult Brain							------------	0.93	0.91
Adult Kidney								------------	0.96
Adult Lung									------------

Supplementary
Figure 4

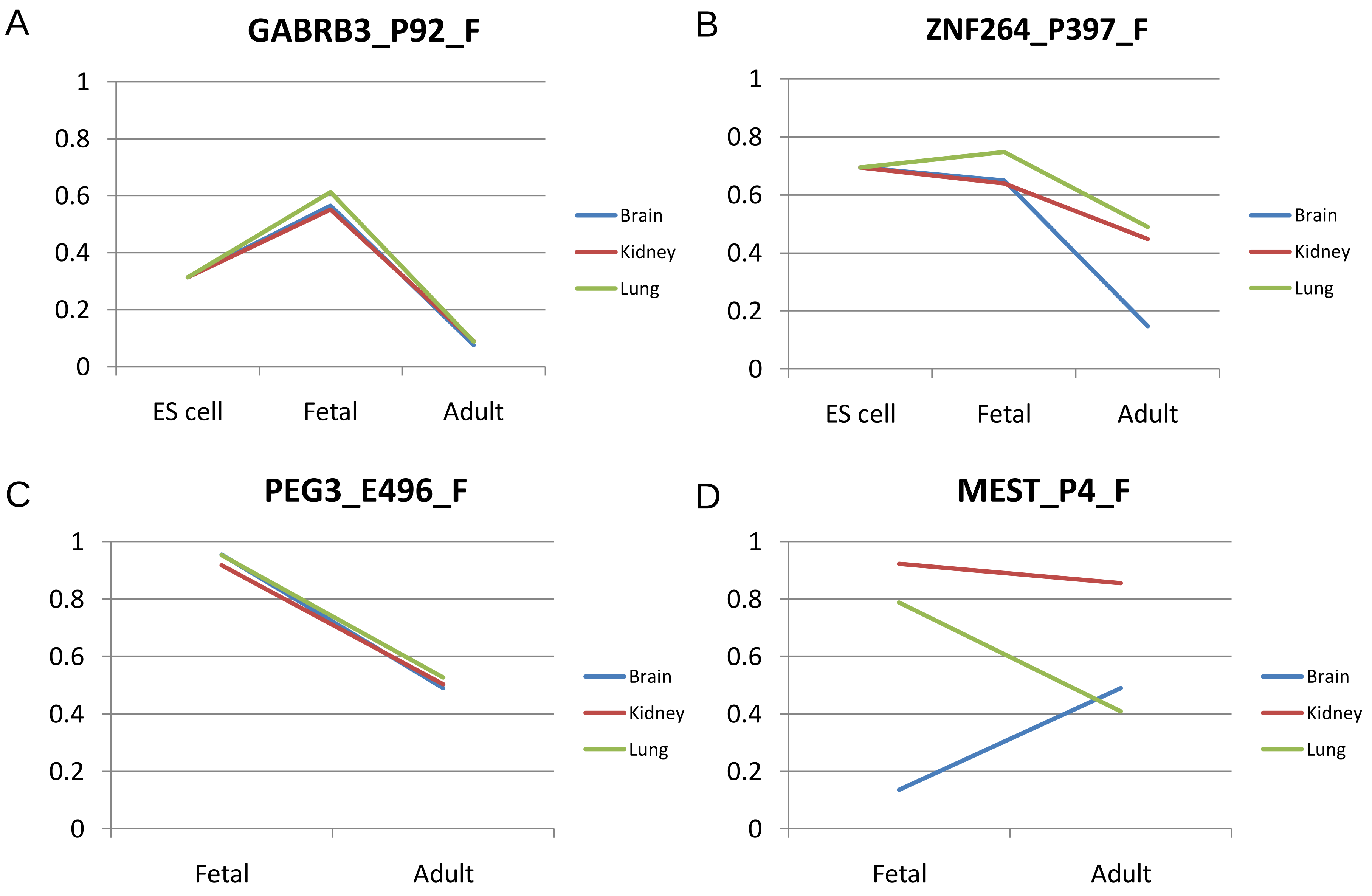

Supplementary Figure 5

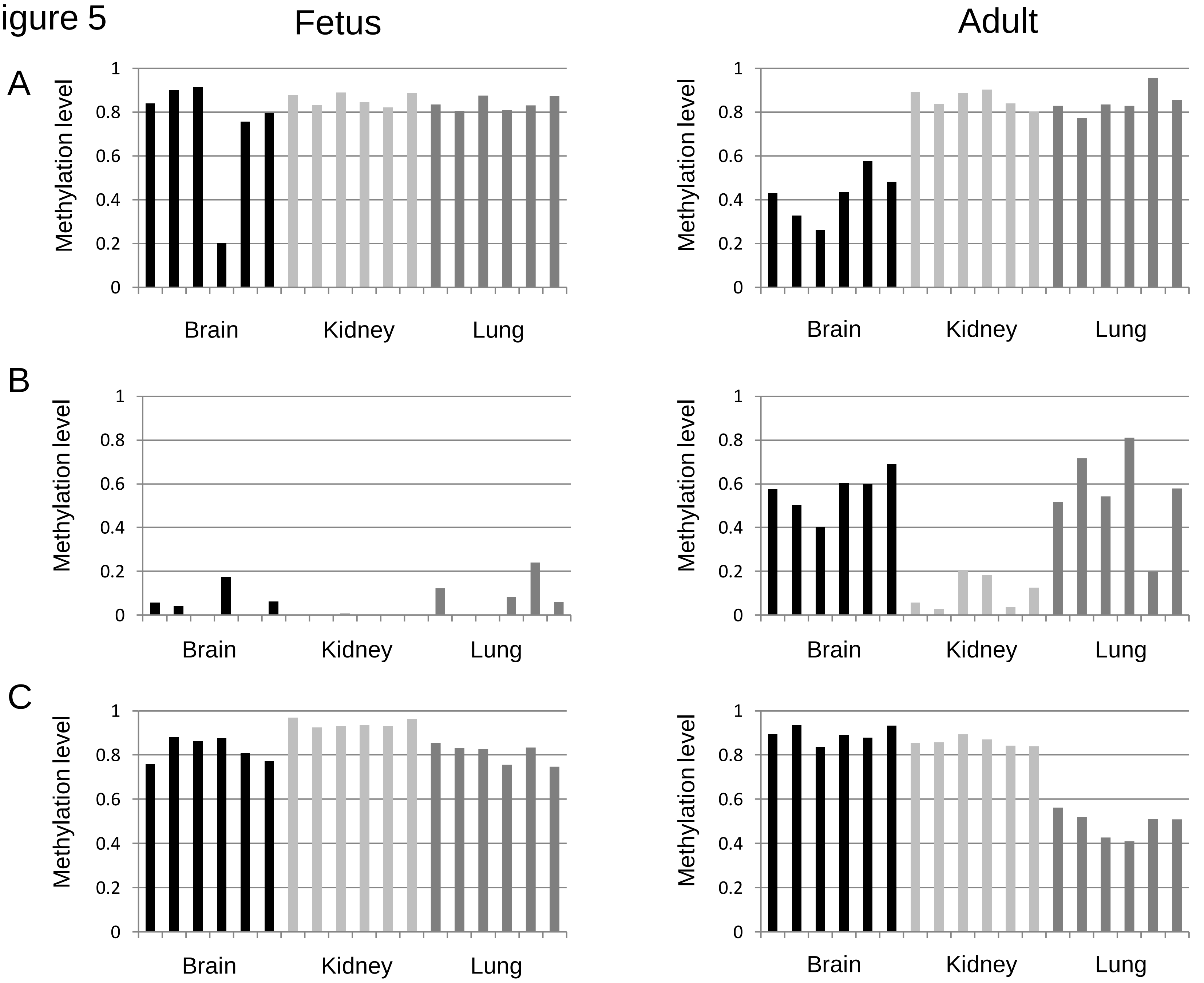

Supplementary Figure 6

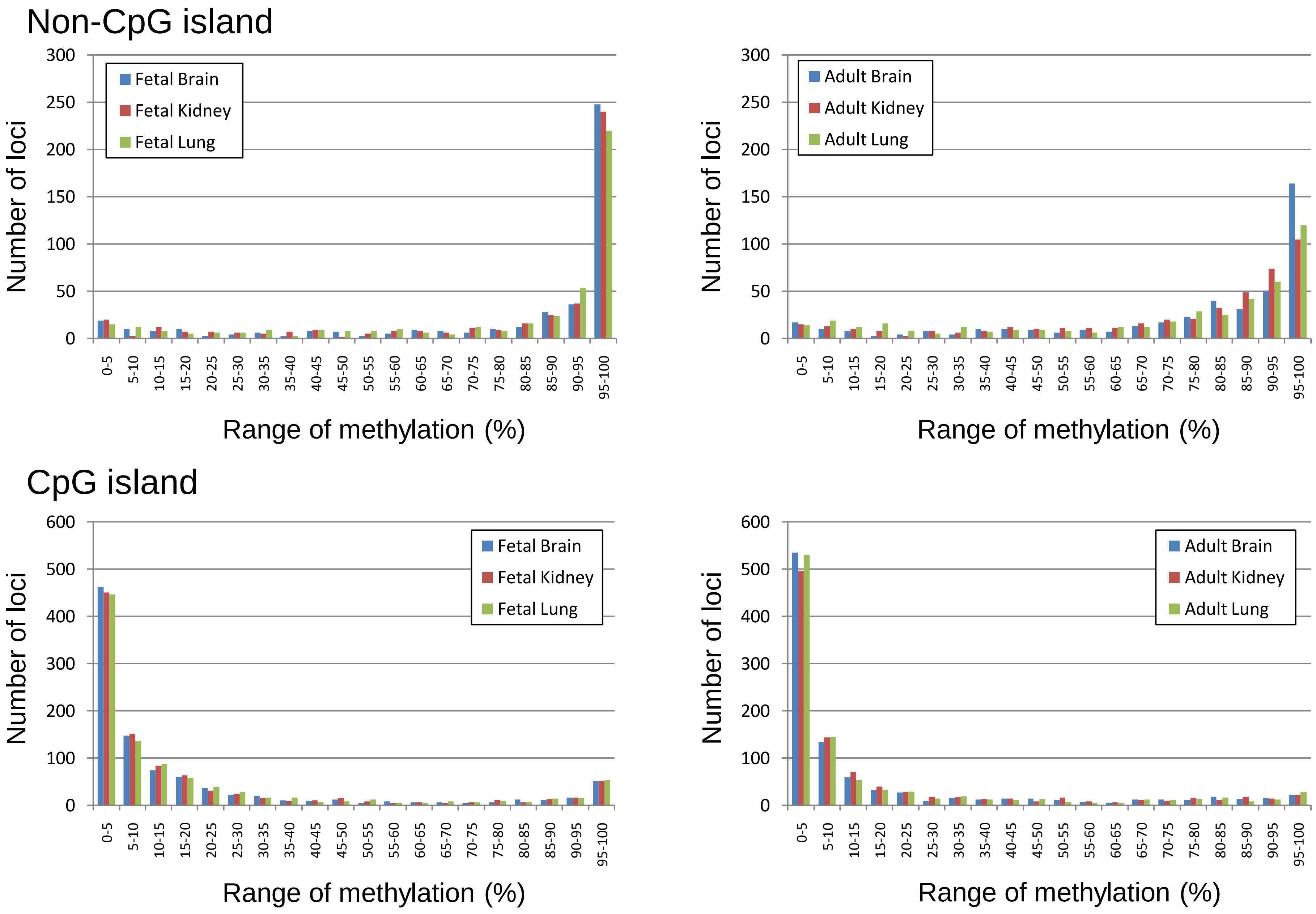

Supplementary Figure 7

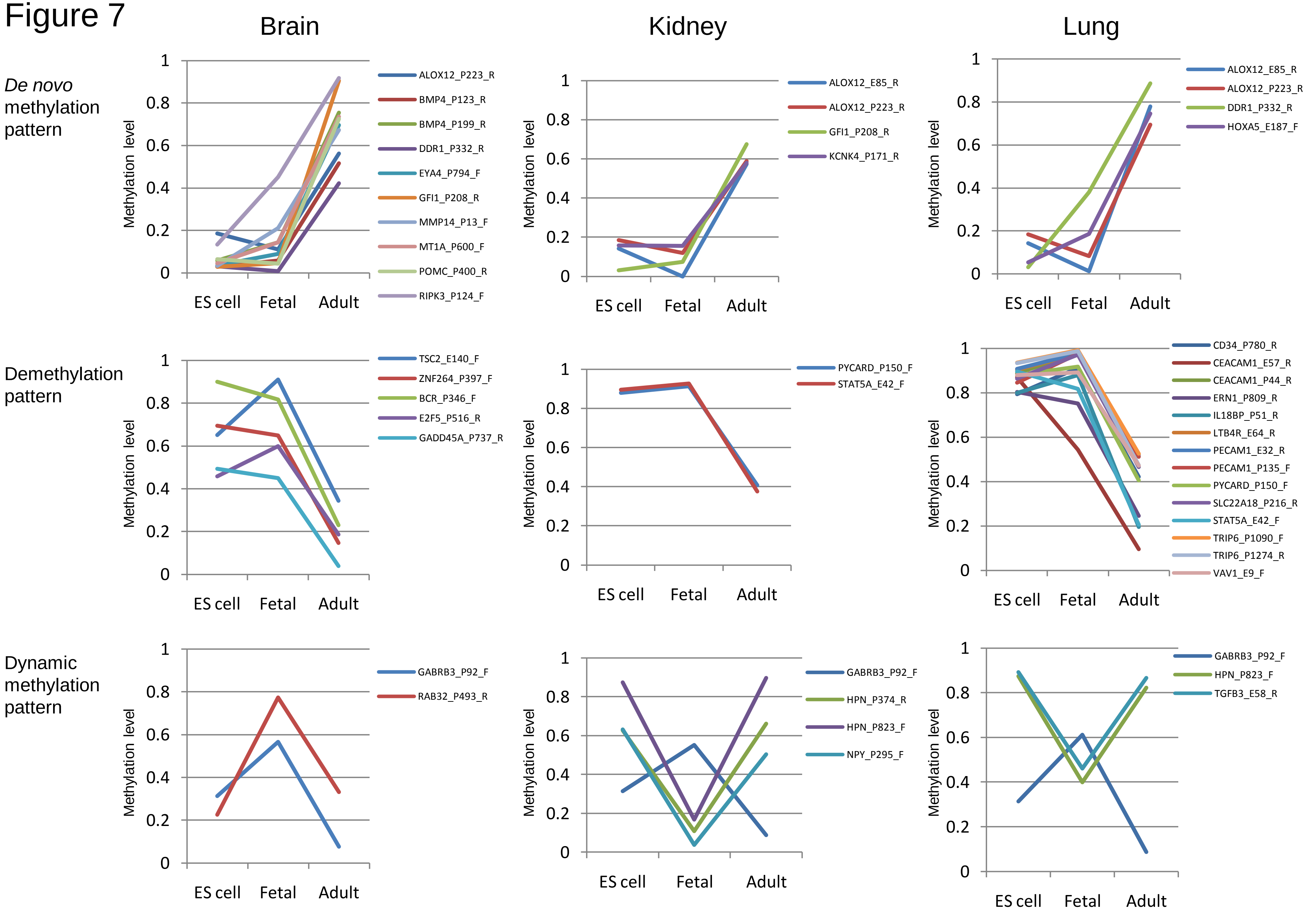

Supplement: Additional file 1 — Figure S1 to S7 [file 1756-8935-4-7-S1.PDF]
